# A novel necroptosis-related gene signature for predict prognosis of glioma based on single-cell and bulk RNA sequencing

**DOI:** 10.3389/fmolb.2022.984712

**Published:** 2022-08-30

**Authors:** Kai Guo, Xinxin Duan, Jiahui Zhao, Boyu Sun, Xiaoming Liu, Zongmao Zhao

**Affiliations:** ^1^ Department of Neurosurgery, The Second Hospital of Hebei Medical University, Shijiazhuang, China; ^2^ Department of Neurosurgery, Affiliated Xing Tai People Hospital of Hebei Medical University, Xingtai, China; ^3^ Department of Oncology, Hebei General Hospital, Shijiazhuang, China; ^4^ Graduate School, North China University of Science and Technology, Tangshan, China; ^5^ Department of Neurology, Beijing Tiantan Hospital, Capital Medical University, Beijing, China

**Keywords:** glioma, necroptosis, prognosis, single-cell sequence, tumor microenvironment, immune checkpoints

## Abstract

**Background:** Glioma is the most fatal neoplasm among the primary intracranial cancers. Necroptosis, a form of programmed cell death, is correlated with tumor progression and immune response. But, the role of necroptosis-related genes (NRGs) in glioma has not been well-uncovered.

**Methods:** Single-cell and bulk RNA sequencing data, obtained from publicly accessed databases, were used to establish a necroptosis-related gene signature for predicting the prognosis of glioma patients. Multiple bioinformatics algorithms were conducted to evaluate the efficacy of the signature. The relative mRNA level of each signature gene was validated by quantitative real-time reverse transcription PCR (qRT-PCR) in glioma cell lines compared to human astrocytes.

**Results:** In this predicted prognosis model, patients with a high risk score showed a shorter overall survival, which was verified in the testing cohorts. The signature risk score was positively related with immune cell infiltration and some immune check points, such as CD276 (B7-H3), CD152 (CTLA-4), CD223 (LAG-3), and CD274 (PD-L1). Single-cell RNA sequencing analysis confirmed that the glioma microenvironment consists of various immune cells with different markers. The eight NRGs of the signature were detected to be expressed in several immune cells. QRT-PCR results verified that all the eight signature genes were differentially expressed between human astrocytes and glioma cells.

**Conclusion:** The eight NRGs correlate with the immune microenvironment of glioma according to our bioinformatics analysis. This necroptosis-related gene signature may evaluate the precise methodology of predicting prognosis of glioma and provide a novel thought in glioma investigation.

## 1 Introduction

Malignant gliomas are the most fatal diseases among the primary intracranial cancers, which were derived from neuronal and glial progenitor cells (Sanai et al., 2005). Gliomas had been classified into four grades (WHO I–IV) in the newest WHO (World Health Organization) 2016 classification of glioma (Wesseling and Capper, 2018). Glioblastoma (WHO IV), as the most aggressive stage, accounts for up to 50% of glioma cases (Ostrom et al., 2015). At present, the standard treatments for glioblastoma (GBM) were recognized as surgical resection, radiotherapy, and temozolomide (TMZ) chemotherapy (Stupp et al., 2005). Emerging evidence suggest that patients could benefit from tumor-treating fields (TTFs) plus temozolomide therapy (Stupp et al., 2015). But, the overall survival (OS) of GBM patients is only 14.6 months even if they accepted the standard treatments (Cloughesy et al., 2014). Nowadays, new molecular subtypes have been widely clinically utilized to predict the prognosis and therapy outcomes of the glioma patients, such as IDH (isocitrate dehydrogenase) mutation and 1p/19q codeletion on chromosomal and 6-O-methylguanine-DNA methyltransferase (*MGMT*) promoter methylation (Lim et al., 2018). These biomarkers were verified to be effective in predicting glioma patients’ outcomes. Nevertheless, due to the heterogeneity of glioma, there is still an urgent need to explore new prediction models.

In 1988, a study showed that the tumor necrosis factor (TNF) could cause a necrotic form of cell death in L-M cells (Laster et al., 1988). Also, this type of cell death was identified as necroptosis in 2005 (Degterev et al., 2005). Cellular morphologies of necroptosis include organelle swelling, progressive translucent cytoplasm, and cell membrane rupture (Vandenabeele et al., 2010). As a form of programmed cell death, necroptosis has been linked to both tumor progression and immune response. Necroptosis played a dual role in tumor, pro-tumorigenic or antineoplastic, which depended on the type of the tumor (Lee et al., 2020; Yan et al., 2022). Furthermore, studies have shown that necroptosis contributes to tumor metastasis (Cai et al., 2016; Strilic et al., 2016). Receptor-interacting serine-threonine kinase 1 (RIPK1), RIPK3, and mixed lineage kinase domain-like (MLKL) were identified as the critical signaling molecules in necroptosis (Zhang et al., 2009; Sun et al., 2012). Additionally, RIPK1/RIPK3 activation was proved to be correlated with the enhancement of antitumor immunity *via* maturated DC and CD8^+^ T cells in the tumor microenvironment (TME) (Park et al., 2021). The necroptosis-related gene (NRG) *PDIA4* was thought to be interrelated with glioma-related immune cells, such as CD8^+^ T cells, Tregs, and eosinophils (Li et al., 2021). Previous research has proven that the mutants of isocitrate dehydrogenase 1 (*IDH1*) can lead to necroptosis resistance by inducing hypermethylation of the RIPK3 promoter (Yang et al., 2017). Moreover, the *IDH1* mutant had been widely recognized as one of the indicators for molecular typing of glioma (Pirozzi and Yan, 2021). Also, *ANAX1*, another NRG, might act as a regulator of cell-mediated immunity in low-grade glioma (LGG) TME by activating T cells (Lin et al., 2021). Celastrol derivatives were verified as novel potential selections in glioma therapy due to their functions in necroptosis (Feng et al., 2022). At present, the concrete mechanism of necroptosis in glioma progression is still undiscovered.

In our study, we established a novel necroptosis-related prognostic signature in the Chinese Glioma Genome Atlas (CGGA) dataset and systematically validated the predictive performance of the signature in The Cancer Genome Atlas (TCGA) dataset. The results revealed that the necroptosis-related signature may serve as an authentic predictor of overall survival, clinical characteristics, immune cell infiltration, and immunotherapy response based on bulk sequencing and single-cell RNA sequencing (scRNA-seq) analysis.

## 2 Materials and methods

### 2.1 Data collection

Expression data and corresponding clinical information of GSE4290 (23 non-tumor and 157 tumor) and GSE16011 (eight non-tumor and 276 tumor) datasets were retrieved from the Gene Expression Omnibus (GEO) database (https://www.ncbi.nlm.nih.gov/geo/). The ‘readr’ and ‘GEOquery’ R packages were used to transform the probe ID into the gene symbol ID. Expression profiling array and clinical data of 475 samples in the Rembrandt microarray were obtained from the CGGA database (http://www.cgga.org.cn/) (the overview of the GEO and Rembrandt database is shown in Table 1). We obtained TCGA (five non-tumor and 592 tumor samples) and Genotype-Tissue Expression (GTEx) (1,152 normal brain tissues) data from UCSC Xena (http://xena.ucsc.edu/). A total of 1,018 glioma patients’ mRNA sequencing and clinical data were retrieved from the CGGA database, including two batches (batch 1: mRNAseq_693 and batch 2: mRNAseq_325). The mRNA sequencing data and clinical information of the samples of 702 patients (LGG: 449 and GBM:143) were obtained from TCGA dataset (https://portal.gdc.cancer.gov/). The overview of patient information is shown in Table 2. Necroptosis-related protein-coding genes were obtained from the GeneCards database (https://www.genecards.org/) website. The genes are summarized in Supplementary Table S1. Glioma scRNA-seq data were retrieved from CGGA including a total of 6,148 cells obtained from biopsies of 13 glioma patients.

**TABLE 1:** Overview of the GEO and Rembrandt database.

Dataset	Citation	Sample
GSE4290	Sun L, Hui AM, Su Q, Vortmeyer A et al. Neuronal and glioma-derived stem cell factor induces angiogenesis within the brain. Cancer Cell 2006 April; 9(4):287–300. PMID: 16616334	Tumor (n = 157)
		Normal (n = 23)
GSE16011	Gravendeel LA, Kouwenhoven MC, Gevaert O, de Rooi JJ et al. Intrinsic gene expression profiles of gliomas are a better predictor of survival than histology. Cancer Res 2009 December 1; 69(23):9065–72. PMID: 19920198	Tumor (n = 276)
		Normal (n = 8)
Rembrandt	Gusev Y, Bhuvaneshwar K, Song L, Zenklusen JC, Fine H, Madhavan S. The REMBRANDT study, a large collection of genomic data from brain cancer patients. PMID: 30106394	Tumor (n = 383)
		Normal (n = 21)

**TABLE 2:** Overview of patient information in the training, testing, and validation cohorts.

Characteristic	Training cohort	Testing cohort	TCGA Validation	p
n	359	356	592	
PRS_type, n (%)				0.215
Primary	229 (32%)	229 (32%)	0 (0%)	
Recurrent	112 (15.7%)	118 (16.5%)	0 (0%)	
Secondary	18 (2.5%)	9 (1.3%)	0 (0%)	
Grade, n (%)				<0.001
WHO II	91 (7%)	95 (7.3%)	211 (16.1%)	
WHO III	116 (8.9%)	116 (8.9%)	238 (18.2%)	
WHO IV	152 (11.6%)	145 (11.1%)	143 (10.9%)	
Gender, n (%)				0.383
Female	142 (10.9%)	159 (12.2%)	248 (19%)	
Male	217 (16.6%)	197 (15.1%)	344 (26.3%)	
IDH_mutation_status, n (%)				0.005
Mutant	196 (15%)	190 (14.5%)	372 (28.5%)	
Wildtype	163 (12.5%)	166 (12.7%)	220 (16.8%)	
1p19q_codeletion_status, n (%)				0.019
Codel	85 (6.5%)	62 (4.7%)	149 (11.4%)	
Non-codel	274 (21%)	294 (22.5%)	443 (33.9%)	
MGMTp_methylation_status, n (%)				0.051
methylated	213 (29.8%)	185 (25.9%)	0 (0%)	
un-methylated	146 (20.4%)	171 (23.9%)	0 (0%)	
Age, median (IQR)	42 (34, 50.5)	43 (36, 53)	47 (34, 59)	<0.001

### 2.2 Identification of necroptosis-related differential expression genes

The RNA-sequencing data of GTEx and TCGA, downloaded from UCSC Xena, were merged and normalized by the R package “limma”. We used the same method to process the expression matrix of the two batches obtained from CGGA. Patients with omitted clinical data were removed.

GSE4290, GSE16011, Rembrandt, and GTEx-TCGA datasets were analyzed by the R package “limma” to identify the differential expression genes (DEGs). The cut-off *p*-value was set at 0.05, and the absolute value of log2 fold change (|LogFC|) was set at 0.5. The intersection of all DEGs was considered DEGs. The common genes between the DEGs and necroptosis genes were included into the following analysis.

NRDEGs were included in univariate analysis to identify prognosis-related genes. The cut-off *p*-value was set at 0.05. Prognosis-related NRDEGs were utilized to construct a protein–protein interaction (PPI) network on STRING (https://cn.string-db.org/). Proteins with no interactions in the network were removed.

### 2.3 Construction of the necroptosis-related prognostic signature

To construct a necroptosis-related signature, least absolute shrinkage and selection operator regression (LASSO-COX) analysis was performed in the CGGA cohort to remove collinearity genes utilizing ‘glmnet’ and ‘survival’ packages. The samples in the CGGA dataset were randomly divided into the training cohort and testing cohort. The risk score of each patient was calculated with the sum of the product of the coefficient value and the expression of genes. The algorithm is demonstrated below:

$$Risk\;score=\overset{n}{\underset{i=1}{\sum }}\left(Coefi*Xi\right)$$

### 2.4 Validation of the NRDEG signature

The differential expression of each gene in the NRDEGs signature between glioma patients and normal samples was obtained from the Gene Expression Profiling Interactive Analysis (GEPIA) database (http://gepia.cancer-pku.cn/).

The TCGA dataset was used as the external validation cohort to perform the algorithm. The median of risk score was considered the cut-off of the high- and low-risk groups.

We performed survival analysis, receiver operating characteristic curve (ROC), and univariate and multivariate analysis to identify the efficiency of the predicted performance of the NRDEG signature. Samples from the two databases (TCGA and CGGA) were divided into subgroups according to their clinical characteristics (such as tumor type (primary or recurrent), grade, female, male, age (Wen and Kesari, 2008), IDH mutation status, 1p/19q codeletion status, and MGMTp meth status). Survival analysis was performed on each subgroup with the “survival” package in training, testing, and validation cohorts. ROC analysis of 1, 3, and 5 year was plotted using the ‘timeROC’ package. Univariate and multivariate analyses were carried out in each cohort by using the package “survival”. Comparison of risk scores in different subgroups was performed in the TCGA dataset.

Therapeutic response information of TCGA patients was retrieved to evaluate the value of the NRDEG signature in predicting the therapeutic response. Available therapeutic response information and drug response were filtered. We grouped the clinical characteristics, titled “measure of response”, in the TCGA dataset. “Progression disease” was input into the “PD” set. The “complete release”, “partial release”, and “stable disease” were input into the “CR/PR/SD” set. Patients with unavailable information were removed.

To estimate the predicted efficiency of the NRDEG signature, the nomogram and calibration curve were constructed using the “rms” package with all clinical features and NRDEG signature in the TCGA cohort.

### 2.5 Gene set enrichment analysis

Gene Set Enrichment Analysis (GSEA) was conducted between high- and low-risk groups (GSEA v4.2.3). Hallmark h. all.v7.5.1 and Kyoto Encyclopedia of Genes and Genomes (KEGG) (c2. cp.kegg.v7.5.1) gene sets were programmed for functional annotation. Results with a normal *p*-value < 0.05 (NOM p-Val) were considered significant.

### 2.6 ScRNA-seq analysis

ScRNA-seq analysis was used to evaluate the expression of the NRDEGs in tumor samples. ‘Seurat’ is an R package for scRNA-seq expression data quality control, normalization, dimensional reduction, and processing. Genes were removed in the condition of expression in less than three cells. Also, we excluded the cells with less than 200 genes. Then, the extreme values of gene expression were removed (nFeature_RNA > 200 & nFeature_RNA < 2500). The expression data were normalized and dimensionally reduced and clustered by the T-SNE method. Cell markers from “CellMarker” (http://bio-bigdata.hrbmu.edu.cn/CellMarker/index.jsp) and previous studies were utilized to annotate the clusters (Supplementary Table S2). The expression of the genes in the NRDEG signature was presented.

### 2.7 Evaluation of immune cell infiltration status

To explore the correlation between risk score and immune cell infiltration, single-sample gene set enrichment analysis (ssGSEA) and CIBERSORTx analysis were performed in the TCGA cohort. Infiltration of 22 kinds of immune cells of patients in low and high clusters was programmed by CIBERSORTx. Patients were clustered into high- and low-immune infiltration groups by ssGSEA. Correlation analysis between Estimation of STromal and Immune cells in MAlignant Tumor tissues using Expression data (ESTIMATE) scores, immune score, stromal score, and tumor purity and risk score was performed. Correlation analysis between the risk score and some immune checkpoints was performed to predict the response to immunotherapy.

### 2.8 Cell culture

The glioma cell lines (U118, U138, LN229, and HS683) and their culture medium (Dulbecco’s modified Eagle medium) were obtained from Procell Life Science & Technology Co., Ltd. (Wuhan, China). HA and its culture medium (Astrocyte Medium) were purchased from ScienCell Research Laboratories, Inc. (San Diego, CA, USA). All the cells were cultured in a sterile cell incubator at 37 °C with 5% CO_2_.

### 2.9 Quantitative real-time reverse transcription PCR

We conducted qRT-PCR to verify the expression of necroptosis-related signature genes in different human glioma cell lines (U118, U138, LN229, and HS683) and human astrocytes (HA). The cells were collected when the density reached 85%–95%. A Superbrilliant TM 6 min High-quality RNA Extraction Kit (Zhongshi Gene Technology, Tianjin, China, Catalog No. ZS-M11005) was used to extract the total RNA of the cells. RNA reverse transcription was performed using the Supersmart TM 6 min first-Strand cDNA Synthesizer Kit (Zhongshi Gene Technology, Tianjin, China, Cat. No. ZS-M14003). The Supersmart 5xFast SYBR Green qPCR Mix Kit (Zhongshi Gene Technology, Tianjin, China, Cat. No. ZS-M13001) was used to perform qRT-PCR to estimate the expression of target genes. The primers were purchased from Sangon Biotech (Shanghai, China) Co., Ltd. The sequence of these primers is listed as follows: *BUB1B* (forward: 5′-AAA​TGA​CCC​TCT​GGA​TGT​TTG​G-3′, reverse: 5′-GCA​TAA​ACG​CCC​TAA​TTT​AAG​CC-3′), *IKBKB* (forward: 5′-GGA​AGT​ACC​TGA​ACC​AGT​TTG​AG-3′, reverse: 5′-GCA​GGA​CGA​TGT​TTT​CTG​GCT-3′), *PDIA4* (forward: 5′-GGC​AGG​CTG​TAG​ACT​ACG​AG-3′, reverse: 5′-TTG​GTC​AAC​ACA​AGC​GTG​ACT-3′), *RCC2* (forward: 5′-AAG​GAG​CGC​GTC​AAA​CTT​GAA-3′, reverse: 5′-GCT​TGC​TGT​TTA​GGC​ACT​TCT​T-3′), *PRL4* (forward: 5′-GCC​TGC​TGT​ATT​CAA​GGC​TC, reverse: 5′-GGT​TGG​TGC​AAA​CAT​TCG​GC), *TXNIP* (forward: 5′-ATA​TGG​GTG​TGT​AGA​CTA​CTG​GG-3′, reverse: 5′-GAC​ATC​CAC​CAG​ATC​CAC​TAC​T-3′), *VIM* (forward: 5′-GAC​GCC​ATC​AAC​ACC​GAG​TT-3′, reverse: 5′-CTT​TGT​CGT​TGG​TTA​GCT​GGT-3′), *ANXA1* (forward: 5′-GAG​GAG​GTT​GTT​TTA​GCT​CTG​C-3′, reverse: 5′-AGC​AAA-GCG​TTC​CGA​AAA​TCT-3′), and *GAPDH* (forward: 5′-GCA​GGG​GGG​AGC​CAA​AAG​GG-3′, reverse: 5′-TGC​CAG​CCC​CAG​CGT​CAA​AG-3′). The 2^-△△^Ct method was used to calculate the results.

### 2.10 Statistical analysis

The analyses were conducted by R software version 4.1.0 (Statistics Department of the University of Auckland) with the newest version of R packages. *p*-value < 0.05 was considered statistically significant.

## 3 Results

### 3.1 Construction of an NRDEG prognostic Signature

**FIGURE 1:**
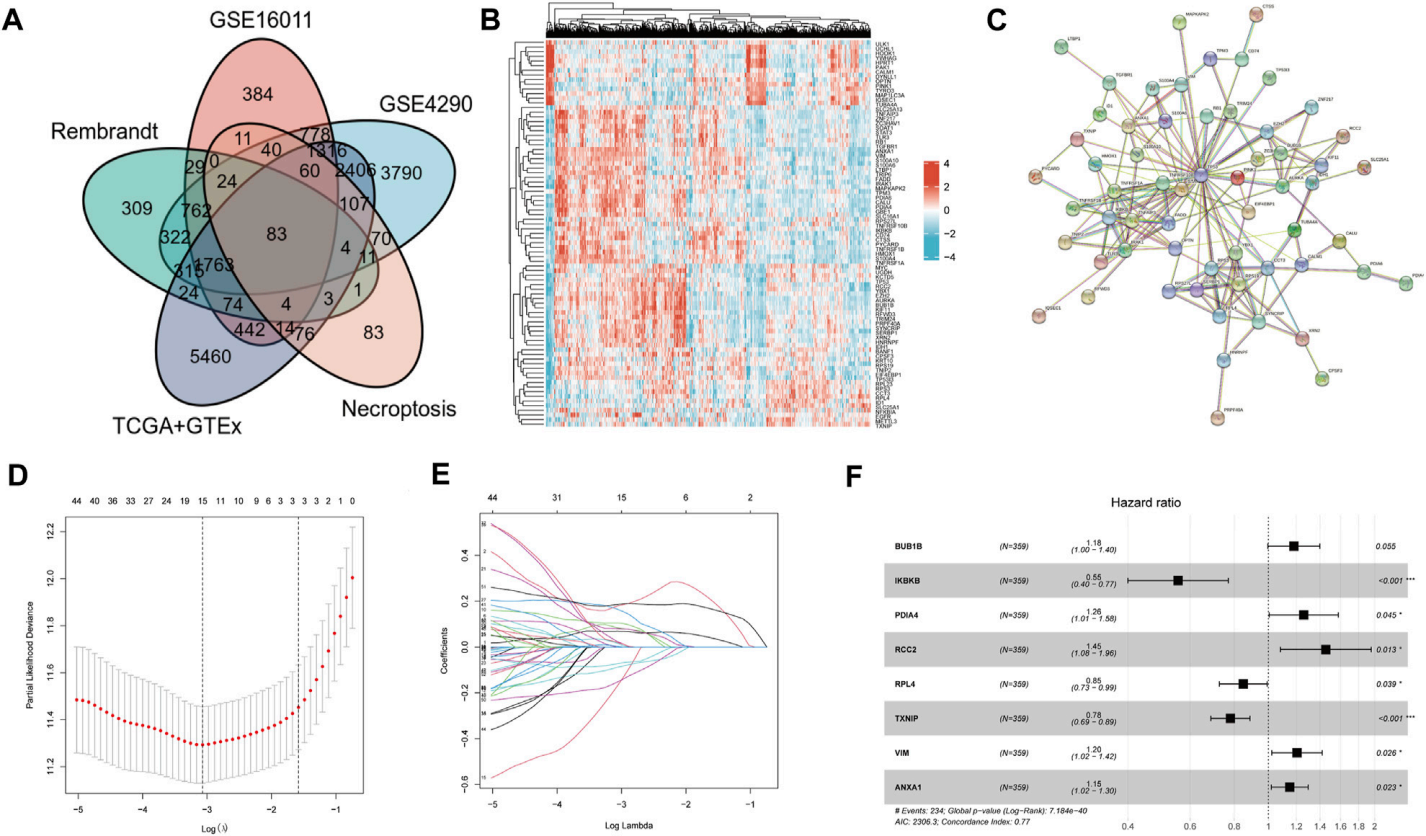
Identification of NRDEGs and construction of NRDEG prognostic signature in glioma. **(A)** Venn diagrams of NRGs and DEGs in four cohorts. **(B)** Heatmap of NRDEGs in the four cohorts (GSE4290, GSE16011, Rembrandt, and TCGA + GTEx). **(C)** PPI network of NRDEGs. **(D)** LASSO regression model. **(E)** Identification of collinearity genes and removal. **(F)** Multivariate Cox regression analysis of the eight NRDEGs in the CGGA database.

DEGs were identified in GSE16011, GSE4290, Rembrandt, and TCGA combined with the GTEx dataset. The threshold was set at |logFC| > 0.5 and *p* < 0.05. The DEGs of GSE16011 (n = 5784), GSE4290 (n = 11851), Rembrandt (n = 3728), and TCGA combined with GTEx (n = 12151) are shown in Supplementary Table S3,S4,S5,S6. The intersection genes of the four cohorts were filtered out for the following analysis. Among the DEGs, 83 genes were NRDEGs (Figure 1A). The heatmap showed the expression profile of the NRDEGs (Figure 1B). Univariate regression analysis was performed in the CGGA cohort. Also, we found that 68 NRDEGs were related to prognosis (Supplementary Table S7). Then, a PPI network was constructed with the 68 genes (Figure 1C). The proteins with no interactions with each other were discarded. In total, 60 coding genes were adopted for subsequent analysis.

Patients in the CGGA cohort were separated into training and testing cohorts randomly. LASSO-COX analysis was performed in the CGGA dataset to remove collinearity genes (Figures 1D,E). The signature was constructed of eight genes: *BUB1B*, *IKBKB*, *PDIA4*, *RCC2*, *RPL4*, *TXNIP*, *VIM*, and *ANXA1* (Figure 1F). All eight genes had a trend of upregulated expression in glioma samples compared to normal brain samples (* represents *p* < 0.05) (Supplementary Figure S1). The risk score was calculated as follows:

Risk score = (0.165860662* *BUB1B* exp) +

(−0.58930583* *IKBKB* exp) +

(0.230473139* *PDIA4* exp) +

(0.374329812* *RCC2* exp) +

(−0.163975013* *RPL4* exp) +

(−0.247830169* *TXNIP* exp) +

(0.186082259* *VIM* exp) +

(0.139160909* *ANXA1* exp).

Patients in the TCGA cohort served as an external validation cohort. The risk score of patients in the TCGA dataset were calculated.

### 3.2 Survival analysis of glioma prognostic risk scores and correlations with clinical and pathological features

**FIGURE 2:**
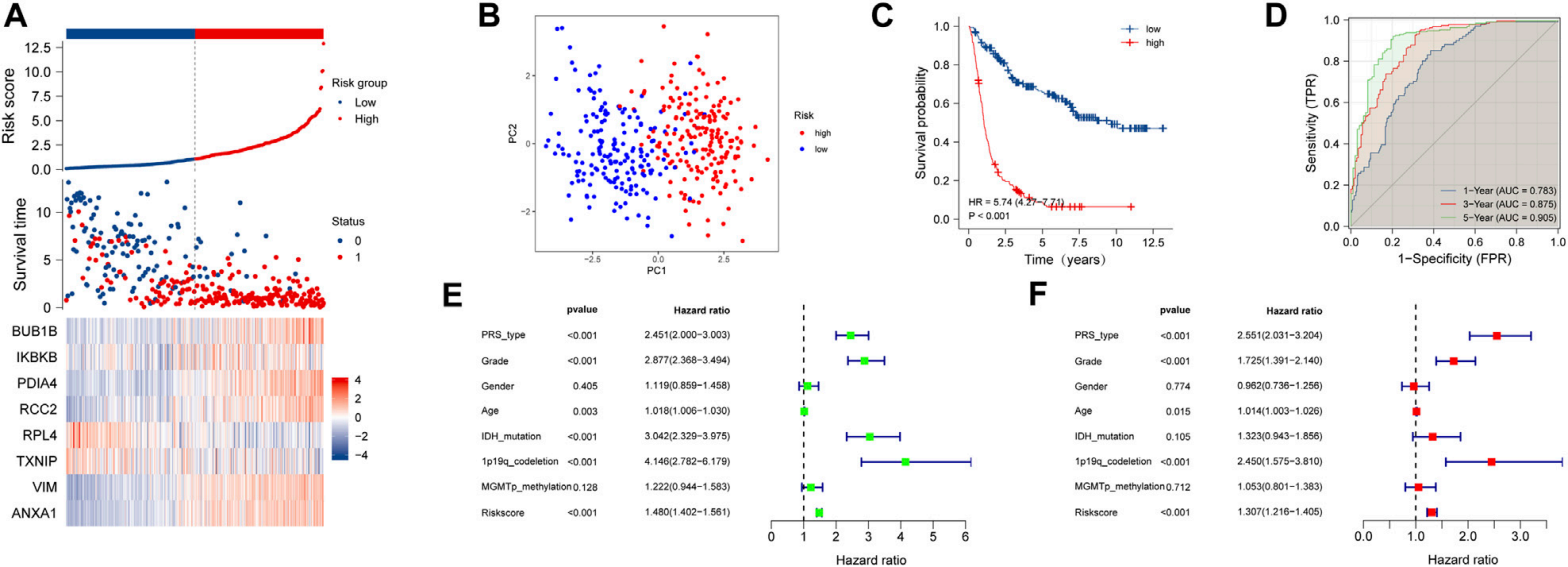
Assessment of the risk score model based on NRDEG prognostic signature in the training cohort **(A)** Risk score, survival distribution of patients with increased risk scores, and expression heatmap of NRDEGs based on risk level. **(B)** PCA of NRDEGs. **(C)** Kaplan–Meier survival of the training cohort. **(D)** AUC of the training cohort (1-, 3-, and 5-year). **(E)** Univariate COX regression analysis of the training cohort. **(F)** Multivariate COX regression analysis of the training cohort.

The results of the training cohort showed that patients in the high-risk group served a worse prognosis than those in the low-risk group (Figure 2A). Principal component analysis (PCA) demonstrated that the necroptosis-related signature was a good indicator in distinguishing the patients with low and high risk (Figure 2B). The survival time of patients with high-risk scores was obviously shorter than that of those with low-risk scores (*p* < 0.001) (Figure 2C). The area under the curve (AUC) of 1, 3, and 5 year in the training cohort was 0.783, 0.875, and 0.905, respectively (Figure 2D). Univariate and multivariate COX regression analyses were proceeded to evaluate the relevance between risk score and prognosis. All the available clinical features were included in the analysis. The thresholds of *p*-value were *p* < 0.001 (Figures 2E,F). These results indicated that our NRDEG signature had a strong efficacy for predicting the prognosis of patients with glioma.

It came to similar conclusions in the testing cohort. The signature was identified as an effective prognostic indicator (Supplementary Figure S2A) and could differentiate the low-risk patients from the high-risk patients (Supplementary Figure S2B). The result of survival analysis between the low- and high-risk groups showed a conspicuous discrimination that high-score patients lived much shorter than the others (Supplementary Figure S2C). The AUC of 1, 3, and 5 year was 0.774, 0.845, and 0.840, respectively (Supplementary Figure S2D). Univariate regression analysis showed that the risk score is a risk factor with statistical significance (*p* < 0.001) (Supplementary Figure S2E). But, it did not exhibit a statistically significant result in the multivariate regression analysis (*p* = 0.89) (Supplementary Figure S2F).

In the TCGA external validation cohort, the signature also had a good performance in predicting the prognosis and discriminating patients between different risk groups (Supplementary Figure S3A,B). The survival analysis showed a distinct difference (*p* < 0.001) between the low- and high-risk groups (Supplementary Figure S3C). The AUC of 1, 3, and 5 year was 0.850, 0902, and 0.832, respectively (Supplementary Figure S3D). The result of univariate regression analysis demonstrated that the NRDEG signature functioned as an adverse prognostic factor (*p* < 0.001) (Supplementary Figure S3E). But, the multivariate regression analysis did not show a statistical criterion result (*p* = 0.472) (Supplementary Figure S3F).

**FIGURE 3:**
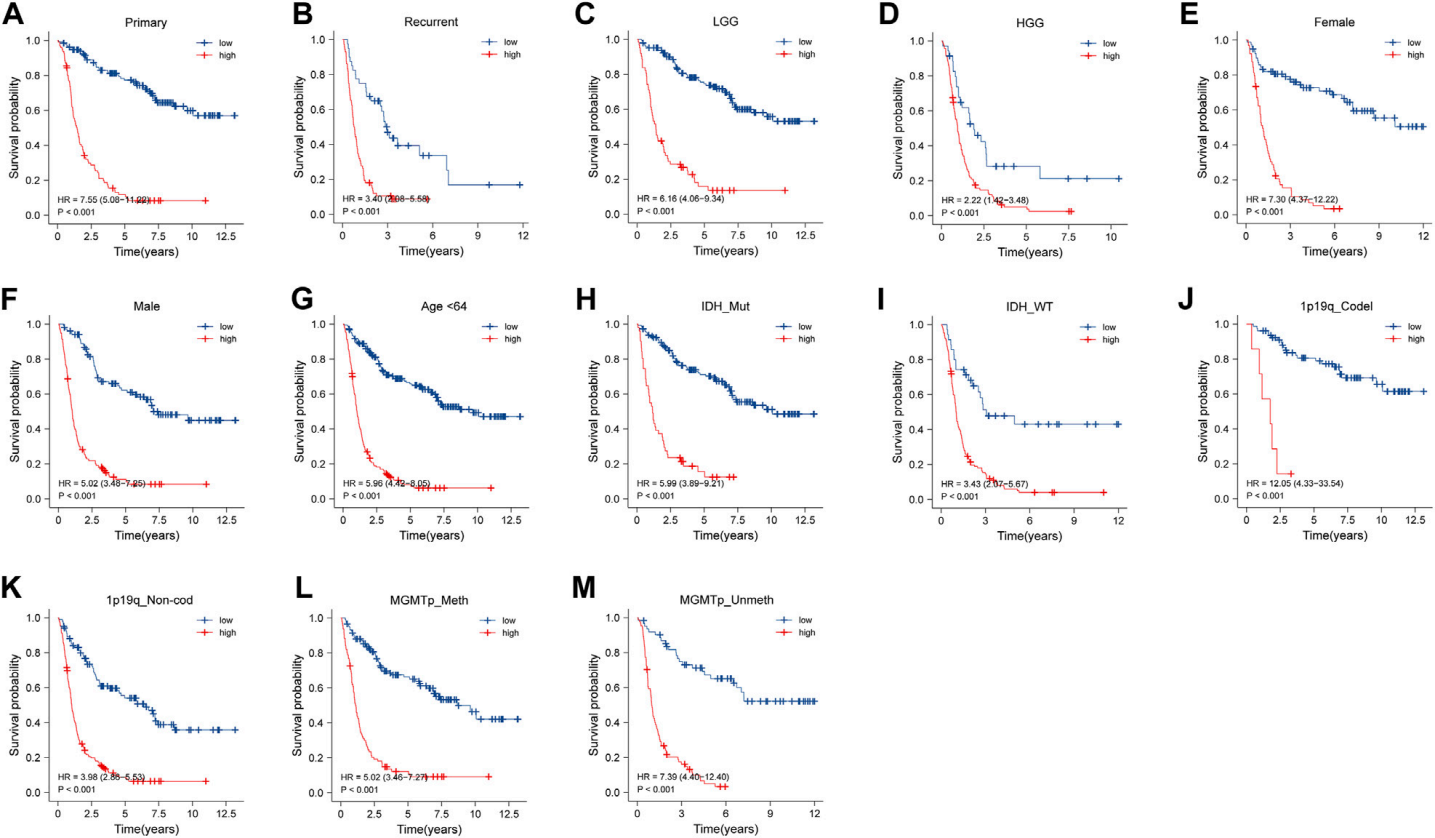
Stratified survival analysis of high- and low-risk score patients in the training cohort by PRS type (primary and recurrence), grade, sex, age, IDH mutant status, 1p/19q codeletion status, and MGMT promoter methylation status **(A–M)**.

Subgroups were divided by the clinical features, including tumor type (primary or recurrent), grade, sex, age, IDH mutation status, 1p/19q codeletion status, and MGMT promoter status. The NRDEG signature had good prediction ability for patients in every subgroup. Patients with high-risk scores had a worse prognosis than those with low-risk scores in the training cohort. The differences between the high- and low-risk groups were statistically significant (*p* < 0.001, separately) (Figure 3A–M).

Survival analysis was conducted in the subgroups in the testing cohort. High-risk patients had a worse prognosis. Also, the results showed that the signature could significantly discriminate the overall survival of patients in the subgroups (*p* < 0.05) (Supplementary Figure S4A–N).

Subgroup survival analysis was carried out in the TCGA external validation cohort as well. In the groups of sex, age, grade, and 1p/19q codeletion status, patients with low risk deserved a higher survival probability (*p* < 0.05) (Supplementary Figure S5A–G). But, no significance was found in the survival probability between the low-risk and high-risk groups in IDH mutation and IDH wildtype subtypes (*p* = 0.125 and *p* = 0.995, respectively) (Supplementary Figure S5H,I).

**FIGURE 4:**
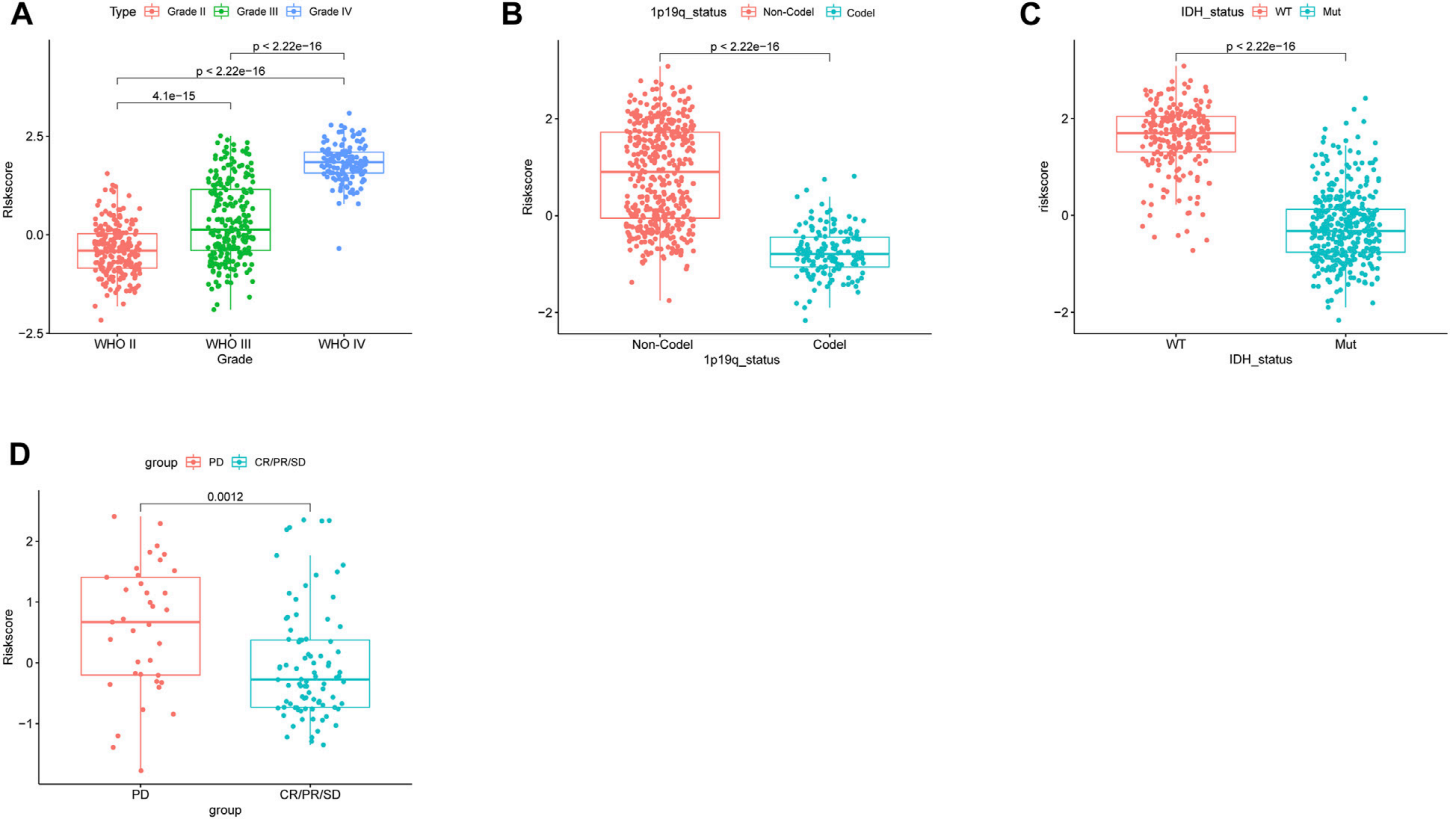
Correlation between the risk score and clinicopathological features in the TCGA database. **(A)** Correlation between the risk score and WHO classification. **(B)** Correlation between the risk score and 1p/19q codeletion status. **(C)** Correlation between the risk score and IDH mutant status. **(D)** Risk score of patients with different responses (PD: progressive disease and CR/PR/SD: complete release/partial release/stable disease) after chemotherapy.

We analyzed the correlation between clinical characteristics and risk scores in the TCGA cohort which contains 592 patients. As the WHO grade of glioma increased, the risk score increased correspondingly (Figure 4A) (*p* < 0.05). Patients with 1p/19q codeletion and IDH mutation had lower risk scores (*p* < 0.05, respectively) (Figures 4B,C).

The NRDEG signature had a good predictive performance for chemotherapy response. Patients in the progressive disease (PD) subgroup had higher risk scores (*p* < 0.05) (Figure 4D).

Above all, the NRDEG signature displayed a good performance in distinguishing between the low- and high-risk groups of glioma patients.

### 3.3 Establishment of a predictive nomogram

**FIGURE 5:**
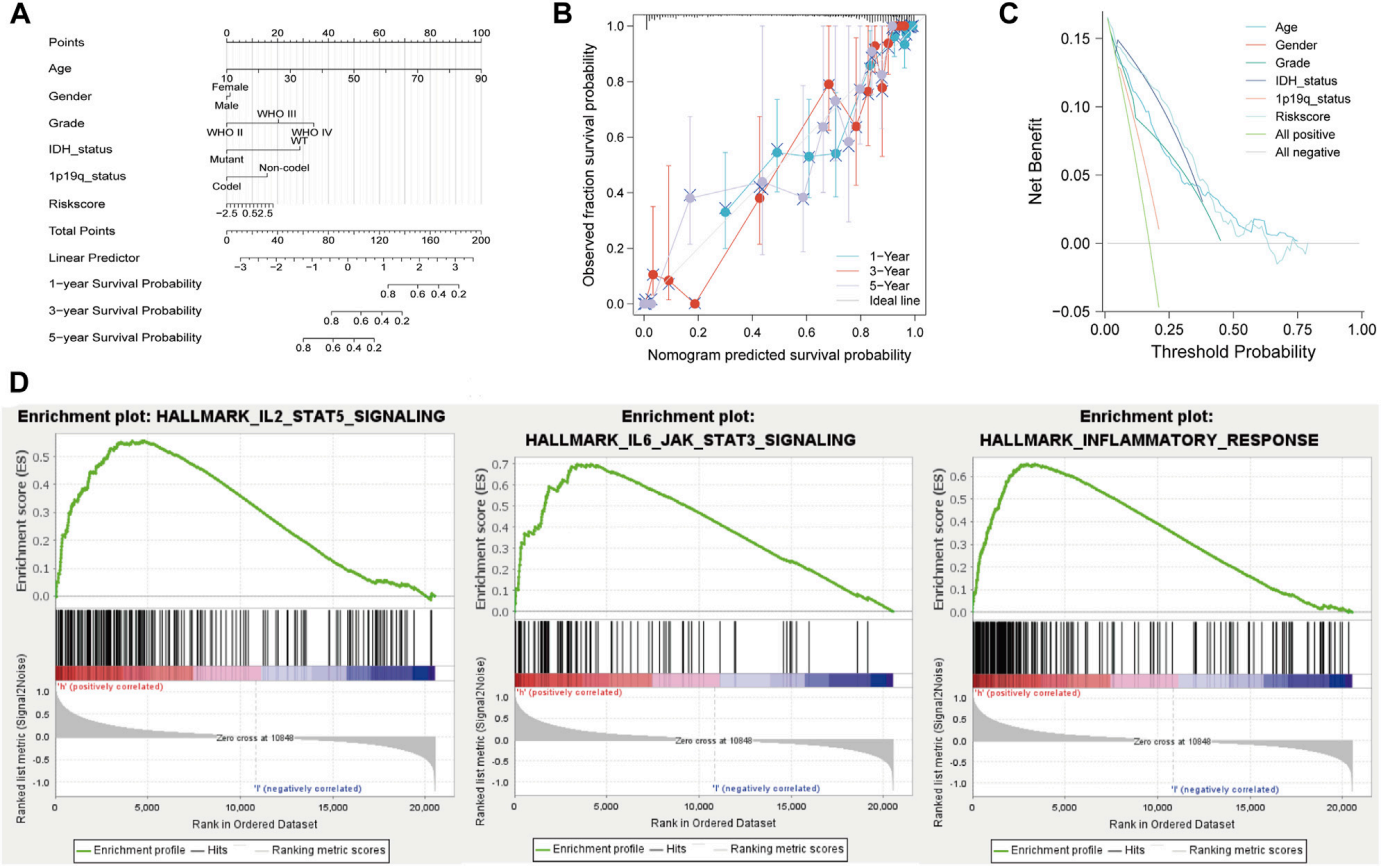
Nomogram and GSEA analysis of the TCGA database. **(A)** Prognostic nomogram. **(B)** Calibration plot of 1-, 3-, and 5-year. **(C)** DCA and **(D)** GSEA of the high- and low-risk score groups. Normal *p*-value < 0.05 (NOM p-Val) were considered significantly.

A nomogram was established including age, sex, grade, IDH status, 1p19q status, and risk score in the TCGA cohort (Figure 5A). The calibration plot showed that NRDEG signature had a good predictive performance for predicting the prognosis of 1-, 3-, and 5-year overall survival (Figure 5B). Decision curve analysis (DCA) clarified that clinical features combined with the signature could predict the prognosis with more sensitivity (Figure 5C). The C-index was 0.863, which illustrates that our predictive nomogram serves as an ideal predictive value.

### 3.4 Gene Set Enrichment Analyses

GSEA results showed that the majority of NRG prognostic signature was involved in immune response-related signals (Figure 5D). The enriched immune response-related signals contain IL-2_STATS signaling, IL-6_STAT3 signaling, and inflammatory response.

### 3.5 ScRNA-seq analysis revealed the tumor microenvironment in glioma

**FIGURE 6:**
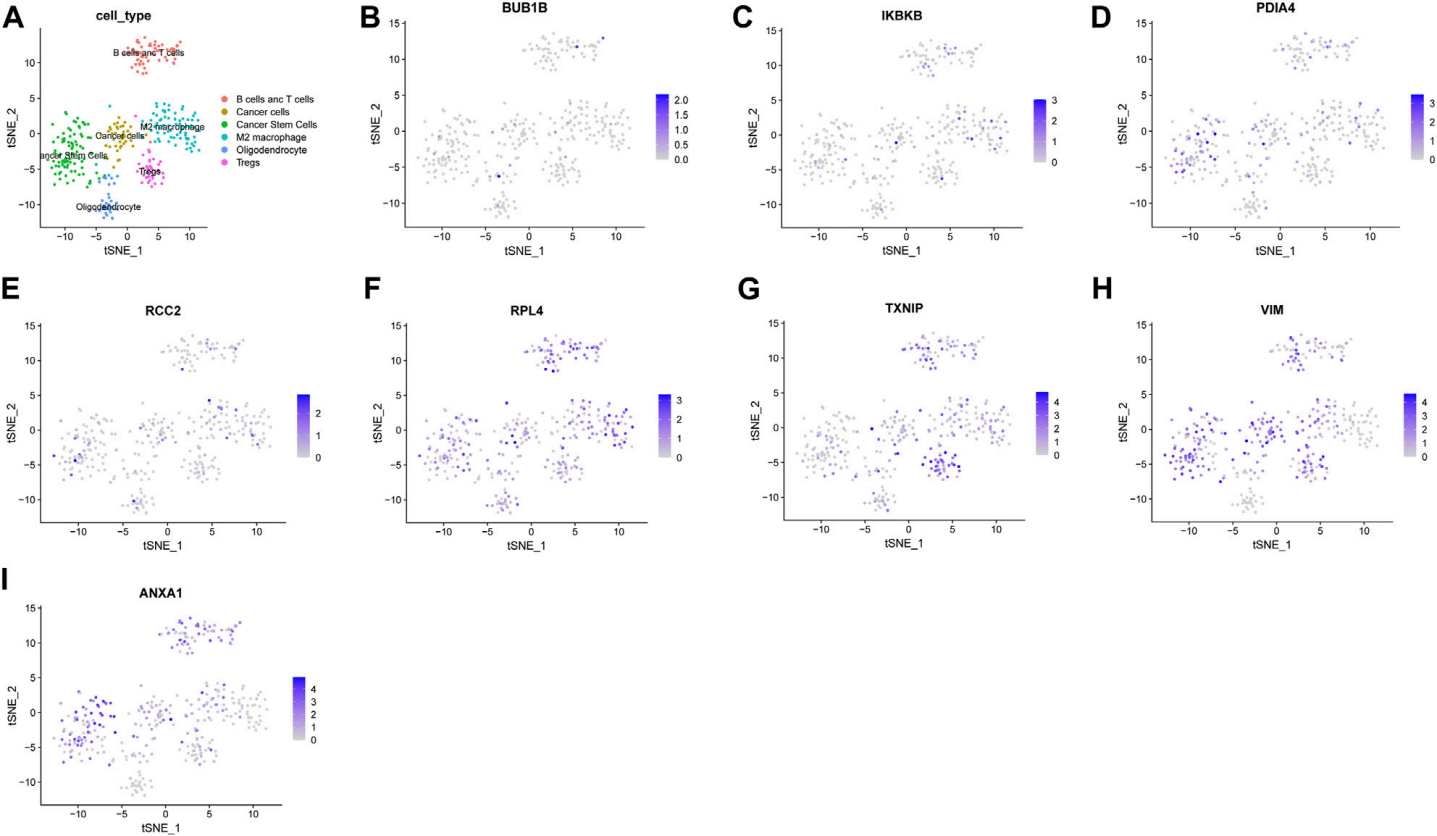
Analysis of the CGGA single-cell RNA sequencing database. **(A)**Annotation of clusters **(B–I)** Expression of NRDEG signature genes by scRNA-sequencing (*BUB1B*, *IKBKB*, *PDIA4*, *RCC2*, *RPL4*, *TXNIP*, *VIM*, and *ANXA1*).

To estimate the tumor microenvironment in glioma patients, scRNA-seq analysis was conducted. CGGA scRNA-seq data contain 6148 cells derived from 13 glioma patients. The overview of the dataset was plotted (Supplementary Figure S6A). After quality control, 22,947 genes and 351 cells were preserved. Variable features were set at 1000 (Supplementary Figure S6B). The top 10 genes from 1,000 highly variable features were plotted (Supplementary Figure S6C). The Jackstraw method was used for dimension reduction. Nine principal components were considered for the following analysis (Supplementary Figure S6D,E). “T-SNE” method was utilized for dimensional reduction analysis. Cells were clustered in six clusters (Supplementary Figure S6F). Markers for annotation of the clusters were obtained from a previous study and “Cellmarker” (http://bio-bigdata.hrbmu.edu.cn/CellMarker/index.jsp). The clusters were annotated (Figure 6A). The expression of the genes in the NRDEG signature was plotted (Figures 6B–I). Genes are expressed in different clusters, especially immune cells. The detailed expression of each gene in different immune cells is shown in Supplementary Figure S7.

### 3.6 Assessment of immune cell infiltration in the tumor microenvironment

**FIGURE 7:**
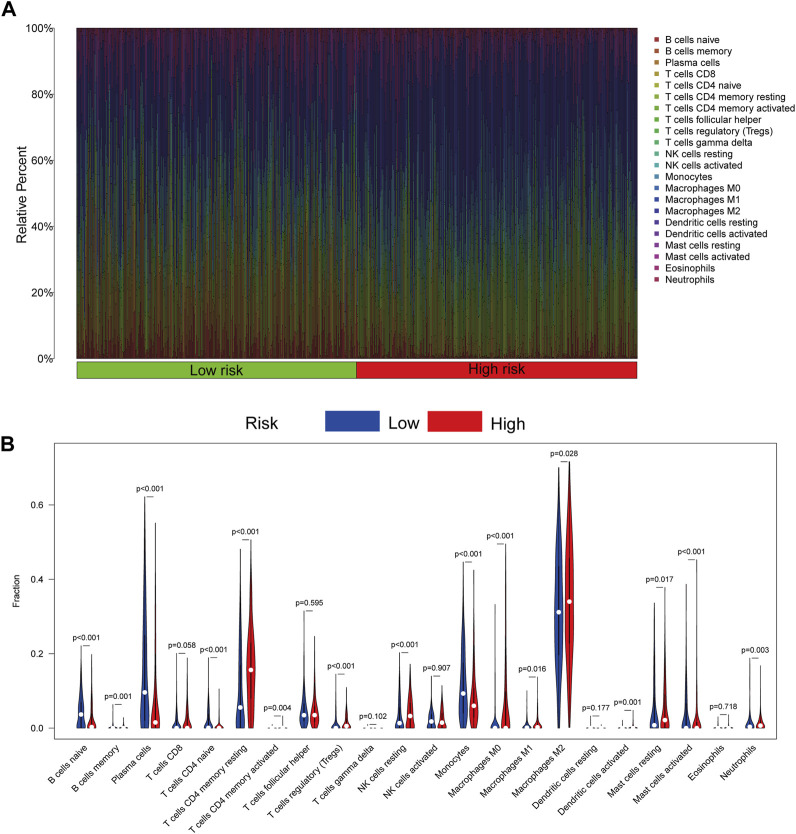
Immune cell infiltration of patients in the TCGA cohort by CIBERSORTx. **(A)** Infiltration of 22 kinds of immune cells from patients in the TCGA cohort. **(B)** Infiltration of 22 kinds of immune cells in patients with high- and low-risk scores. *p* < 0.05 was considered a threshold of statistical significance.

CIBERSORTx was utilized to assess the immune infiltration of patients in the TCGA cohort. The percentage of immune cells is displayed in Figure 7A. Patients in the high-risk group had more regulatory T-cell (Tregs) (*p* < 0.001), M2 macrophage (*p* = 0.028), resting NK cell (*p* < 0.001), and resting mast cell (*p* = 0.017) infiltration (Figure 7B).

**FIGURE 8:**
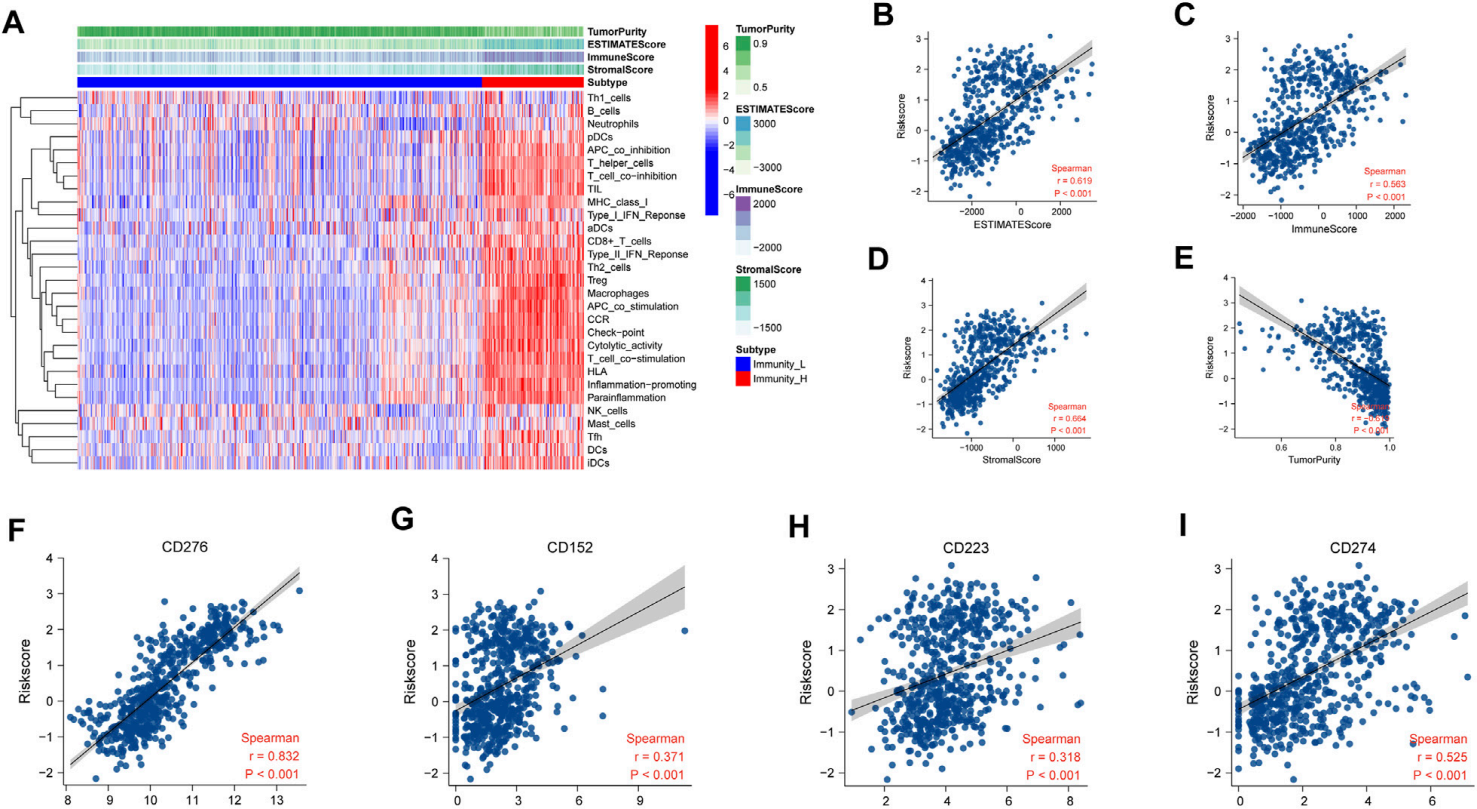
Assessment of immune infiltration in TCGA. **(A)** Heatmap of ssGSEA scores of immune cells and functions. **(B–E)** Relationship of risk score between ESTIMATE score (r = 0.619, *p* < 0.001), immune score (r = 0.563, *p* < 0.001), stromal score (r = 0.664, *p* < 0.001), and tumor purity (r = -0.679, *p* < 0.001). **(F–I)** Relationship of risk score between immune check points. CD276 (r = 0.832, *p* < 0.001), CD152 (r = 0.371, *p* < 0.001), CD223 (r = 0.318, *p* < 0.001), and CD274 (r = 0.525, *p* < 0.001). The relative mRNA expression of necroptosis-related signature genes in U118, U138, LN229, and HS683 glioma cell lines compared to HA.

To validate the results, we used ESTIMATE to evaluate the purity of tumor tissues (Figure 8A). The risk score is positively related with the ESTIMATE score (r = 0.619, *p* < 0.001), immune score (r = 0.563, *p* < 0.001), and stromal score (r = 0.664, *p* < 0.001), but not tumor purity (r = -0.679, *p* < 0.001) (Figures 8B–E). Patients with high-risk scores had more immune cell infiltration than low-risk patients.

To predict immunotherapy response, the relevance between risk score and some immune checkpoints was programmed. The NRDEG risk score is positively correlated with CD276 (r = 0.832, *p* < 0.001), CD152 (r = 0.371, *p* < 0.001), CD223(r = 0.318, *p* < 0.001), and CD274 (r = 0.525, *p* < 0.001) (Figures 8F–I).

### 3.7 Validation of necroptosis-related signature genes in human glioma cell lines

**FIGURE 9:**
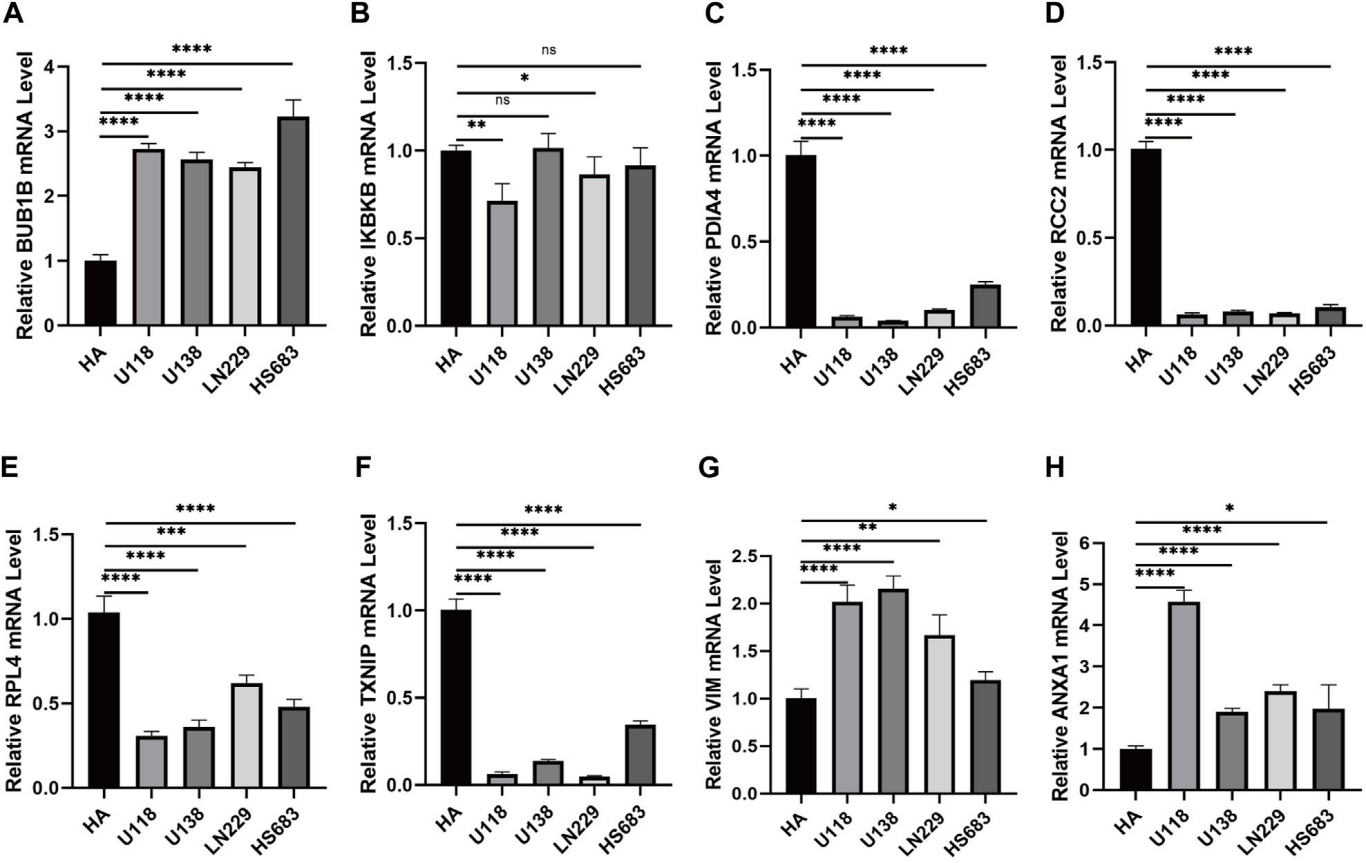
**(A)** Relative mRNA expression of *BUB1B* in different glioma cell lines. **(B)** Relative expression of *IKBKB* mRNA in different glioma cell lines. **(C)** Relative expression of *PDIA4* mRNA in different glioma cell lines. **(D)** Relative expression of *RCC2* mRNA in different glioma cell lines. **(E)** Relative expression of *RPL4* mRNA in different glioma cell lines. **(F)** Relative expression of *TXNIP* mRNA in different glioma cell lines. **(G)** Relative expression of *VIM* mRNA in different glioma cell lines. **(H)** Relative expression of *ANXA1* mRNA in different glioma cell lines. *p < 0.05, **p < 0.01, ***p < 0.001, and ****p < 0.0001.

QRT-PCR was used to verify the expression of necroptosis-related signature genes. The results showed that *BUB1B* was overexpressed in U118, U138, LN229, and HS683 cell lines than HA (Figure 9A). *IKBKB* was expressed at low levels in U118 and LN229. Also, there was no significant difference observed in U138 and HS683 (Figure 9B). The mRNA levels of *PDIA4*, *RCC2*, *PRL4*, and *TXNIP* in U118, U138, LN229, and HS683, respectively, were notably lower than in HA (Figures 9C–F). The relative mRNA expression of *VIM* and *ANXA1* was observably higher than that of HA (Figures 9G,H).

## 4 Discussion

Gliomas are recognized as the most malignant primary tumor in the intracranial central nerve system. Epidemiological data showed that gliomas constituted 30% of the primary brain neoplasms. Furthermore, the proportion of gliomas in malignant brain tumors has reached 80% (Weller et al., 2015). Due to the malignancy and recurrence rate of gliomas, the patients tend to have a poor prognosis, though they have accepted comprehensive treatment, including surgical resection, radiotherapy, chemotherapy, TTF, and immunotherapy (Park et al., 2020). Despite that gliomas cannot be cured completely, the diagnosis and treatment of gliomas is continually making advancements.

The concept of necroptosis had been introduced in 2010 (Vandenabeele et al., 2010). It is a form of regulated cell death that relies on RIPK1, RIPK3, and MLKL (Galluzzi et al., 2017). Necrostatin-1 (Nec-1) was identified as the first necroptosis inhibitor by inhibiting the activity of RIPK1 (Degterev et al., 2008). The tumor necrosis factor receptor superfamily was considered the primary activator of necroptosis based on plenty of reports (Gong et al., 2019). Additionally, T-cell receptors, pattern recognition receptors, and multiple chemotherapeutic drugs were also identified as the stimulator of necroptosis (Lalaoui et al., 2015). Necroptosis played a complex role in the tumor, both in repressing tumor progression and promoting tumorigenesis and metastasis (Lee et al., 2020; Yan et al., 2022). It depended on the type of cancer. In recent years, the progress of bulk RNA-seq and scRNA-seq has led to identification of alteration of gene and the immune microenvironment in glioma. Specific algorithms, based on the sequencing data, were developed to make a more precise diagnosis in glioma. Different types of signatures had been established to predict the prognosis of glioma patients. A necroptosis-related signature had been constructed based on bulk RNA-seq analysis (Zhou et al., 2022). We thought it is necessary to construct an NRDEG signature based on more details. So, we performed the present study based on bulk RNA-seq and scRNA-seq analysis in more databases to predict the prognosis of glioma patients. Also, our NRG prognostic signature showed a new vision of immune cell infiltration in the glioma microenvironment.

We constructed an eight NRG prognostic signature of patients with glioma. *BUB1B*, one of the components of the mitotic checkpoint complex, plays an essential role in maintaining chromosome stability (Musacchio and Salmon, 2007). It had been experimentally proven that *BUB1B* is overexpressed in glioma and associated with tumorigenicity and radio-resistance of glioma (Ma et al., 2017). The NF-κB pathway had been verified to be overactive in the formation of glioma (Cahill et al., 2016). *IKBKB* participated in blocking the NF-κB signaling pathway by phosphorylating IκB (Perkins, 2007). PDIA4 is a member of the protein disulfide isomerase (PDI) family, and research studies had manifested that *PDIA4* could induce platelet activation and participate in endoplasmic reticulum stress response. Our research demonstrated that *PDIA4* is distinctly low-expressed in glioma cells. Regulator of chromosome condensation 2 (*RCC2*) is essential in regulating chromosome segregation (Mollinari et al., 2003). A previous report suggested that *RCC2* can promote glioma cell proliferation and radio-resistance (Yu et al., 2019). But, there was a lack of experimental validation of the *RCC2* mRNA levels between astrocyte cells and glioma cells in the aforementioned research. Studies indicated that RPL4 participated in regulating tumor cell proliferation (Yang et al., 2019; Wang W. et al., 2021). Thioredoxin-interacting protein (*TXNIP*) was reported as a tumor-suppressor gene in glioma (Zhang et al., 2017). VIM had been identified as a potential biopsy marker for glioma in several bioinformatics analyses (Gao et al., 2019; Wang J. J. et al., 2021; Herrera-Oropeza et al., 2021). Also, our results corroborated these analyses. *ANXA1* had just been reported as a prognostic and immune microenvironmental marker in glioma (Lin et al., 2021; Qian et al., 2021).

The eight genes of the signature were shown to be overexpressed in GBM and LGG compared to normal brain tissues according to the bioinformatics analysis of the TCGA + GTEx cohort (Supplementary Figure S1). But, the RT-qPCR results showed that five genes (*IKBKB*, *PDIA4*, *RCC2*, *RPL4*, and *TXNIP*) were low expressed in glioma cell lines compared to HA (Figure 9). There are several possible reasons to explain the opposite trend. First, the sources of results were different. The RNA-seq data were obtained from glioma samples, which contain glioma cells, immune cells, neurons, normal glial cells, and so on. The glioma samples were complexes of multiple types of cells. The qRT-PCR results were obtained from glioma cell lines without any other types of cells. Additionally, glioma cell lines often lose some biological characteristics due to long-term culture *in vitro*. So, the different trend of the five genes presented in RNA-seq and qRT-PCR results is understandable.

Research studies demonstrated that immune cell infiltration is correlated with the prognosis of cancer patients (Schreiber et al., 2011). The particularity of the tumor microenvironment in glioma is attributed to the cellular heterogeneity in the central nervous system and infiltration of abundant immune cells (Gieryng et al., 2017). Analysis of scRNA-seq revealed that the component of the microenvironment contains various immune cells with different markers. The eight NRDEG signature genes were also expressed in several immune cells. Then, we evaluated the infiltration in glioma by ssGSEA and CIBERSORTx in bulk sequencing. The risk score was positively related to immune cell infiltration. The risk score of our signature was positively related to some immune check points, such as CD276 (B7-H3), CD152 (CTLA-4), CD223 (LAG-3), and CD274 (PD-L1). Previous studies had proven that patients with high expression of the immune check points could benefit from immune check point blockade therapy (Walker and Sansom, 2011; Yi et al., 2018; Wang et al., 2019; Kontos et al., 2021). Patients with high NRDEG risk scores may benefit from immunotherapy. The GSEA results manifested that these eight NRDEGs were enriched in the immune response-related signals. More immunotherapy studies aimed at glioma are needed.

Infiltration of various immune cells has been identified as associated with the prognosis of patients with cancer. It has been confirmed that M2 macrophages can promote tumorigenesis and metastasis (Pan et al., 2020). Similarly, resting memory CD4^+^ T cells and resting mast cells have been found negatively correlated with prognosis (Liu et al., 2021). Furthermore, regulatory T cells (Tregs) can establish an immune suppressor microenvironment (Farhood et al., 2019). In the present study, patients with high-risk scores had increased M2 macrophage, Tregs, resting memory CD4^+^ T cell, and resting mast cell infiltration. Patients in the high-risk score group showed poor OS and immunotherapy resistance, which is consistent with our assessment of immune cell infiltration in the TME. It indicated that our NRDEG signature could quite well assess the infiltration of immune cells and is a robust prognostic signature.

## 5 Conclusion

A reliable NRDEG signature had been established to predict the prognosis of glioma patients. Furthermore, the signature could predict the immunotherapy response of patients with glioma. Also, the signature may be a promising indicator for predicting the response of immunotherapy and immune cell infiltration.

## Data Availability

The original contributions presented in the study are included in the article/Supplementary Material; further inquiries can be directed to the corresponding author.

## Supplementary material

Supplementary Figure S1 (A–H) Expression of the prognostic signature genes between glioma samples and normal samples in TCGA + GTEx from GEPIA. (GBM: glioblastoma and LGG: lower grade glioma) (*: *P* < 0.05).

Supplementary Figure S2 Assessment of the risk score model based on NRDEG prognostic signature in the testing cohort. **(A)** Risk score, survival distribution of patients with increased risk scores, and expression heatmap of NRDEGs based on the risk level. **(B)** PCA of NRDEGs. **(C)** Kaplan–Meier survival of the testing cohort. **(D)** Area under the curve (AUC) of the testing cohort (1-, 3-, and 5-year). **(E)** Univariate COX regression analysis of the testing cohort. **(F)** Multivariate COX regression analysis of the testing cohort.

Supplementary Figure S3 Validation of the risk score model based on NRDEG prognostic signature in the TCGA external validation cohort. **(A)** Risk score, survival distribution of patients with increased risk scores, and expression heatmap of NRDEGs based on risk level. **(B)** PCA of NRDEGs. **(C)** Kaplan–Meier survival of the validation cohort. **(D)** AUC of the validation cohort (1-, 3-, and 5-year). **(E)** Univariate COX regression analysis of the validation cohort. **(F)** Multivariate COX regression analysis of the validation cohort.

Supplementary Figure S4 Stratified survival analysis of high- and low-risk score patients in the testing cohort, by PRS type (primary, recurrence, and secondary), grade, sex, age, IDH mutant status, 1p19q codeletion status, and MGMT promoter status (A–N).

Supplementary Figure S5 Stratified survival analysis of high- and low-risk score patients in the TCGA external validation cohort. Stratified survival analysis of high- and low-risk score patients in the testing cohort by sex, age, grade, 1p19q codeletion status, and IDH mutant status (A–I).

Supplementary Figure S6 Characteristics of the CGGA scRNA-seq data. **(A)**Violin plot of samples in the CGGA scRNA-seq dataset. **(B)** Identification of 1,000 high variable expressed genes. **(C)** Top 10 high variable expressed genes. **(D)** Identification of linear reduction dimension. **(E)** Scatter plot of linear reduction dimension. **(F)** Clustering of tumor cells by the T-SNE method.

Supplementary Figure S7 Detail expression of each gene in different immune cells.

Supplementary Table S1 Necroptosis-related protein coding genes.

Supplementary Table S2 Cell markers from “Cellmarker”.

Supplementary Table S3 DEGs of GSE16011.

Supplementary Table S4 DEGs of GSE4290.

Supplementary Table S5 DEGs of Rembrandt.

Supplementary Table S6 DEGs of TCGA combined with GTEx.

Supplementary Table S7 Prognosis-related 68 NRDEGs.

## Glossary

AUC: Area under the curve
CGGA: Chinese Glioma Genome Atlas
DCA: Decision curve analysis
DEGs: Differential expression genes
ESTIMATE: Estimation of STromal and Immune cells in MAlignant Tumor tissues using Expression data
GBM: Glioblastoma
GEO: Gene Expression Omnibus
GEPIA: Gene Expression Profiling Interactive Analysis
GSEA: Gene Set Enrichment Analysis
GTEx: Genotype-Tissue Expression
HA: Human astrocytes
IDH: Isocitrate dehydrogenase
IDH1: Isocitrate dehydrogenase 1
LASSO-COX: Least absolute shrinkage and selection operator regression
LGG: Low-grade glioma
|LogFC|: Log2 fold change
MGMT: 6-O-methylguanine-DNA methyltransferase
NRDEGs: Necroptosis-related differential expression genes
NRGs: Necroptosis-related genes
OS: Overall survival
PPI: Protein–protein interaction
qRT-PCR: Quantitative real-time reverse transcription PCR
ROC: Receiver operating characteristic curve
scRNA-seq: Single-cell RNA sequencing
ssGSEA: Single-sample gene set enrichment analysis
TCGA: The Cancer Genome Atlas
TME: Tumor microenvironment
TMZ: Temozolomide
TNF: Tumor necrosis factor
TTF: Tumor-treating fields
WHO: World Health Organization
