# Supplementary figures and images for: A novel necroptosis-related gene signature for predict prognosis of glioma based on single-cell and bulk RNA sequencing
Source: Front Mol Biosci. 2022 Aug 30;9:984712. doi: 10.3389/fmolb.2022.984712 (PMC9469195; doi:10.3389/fmolb.2022.984712)

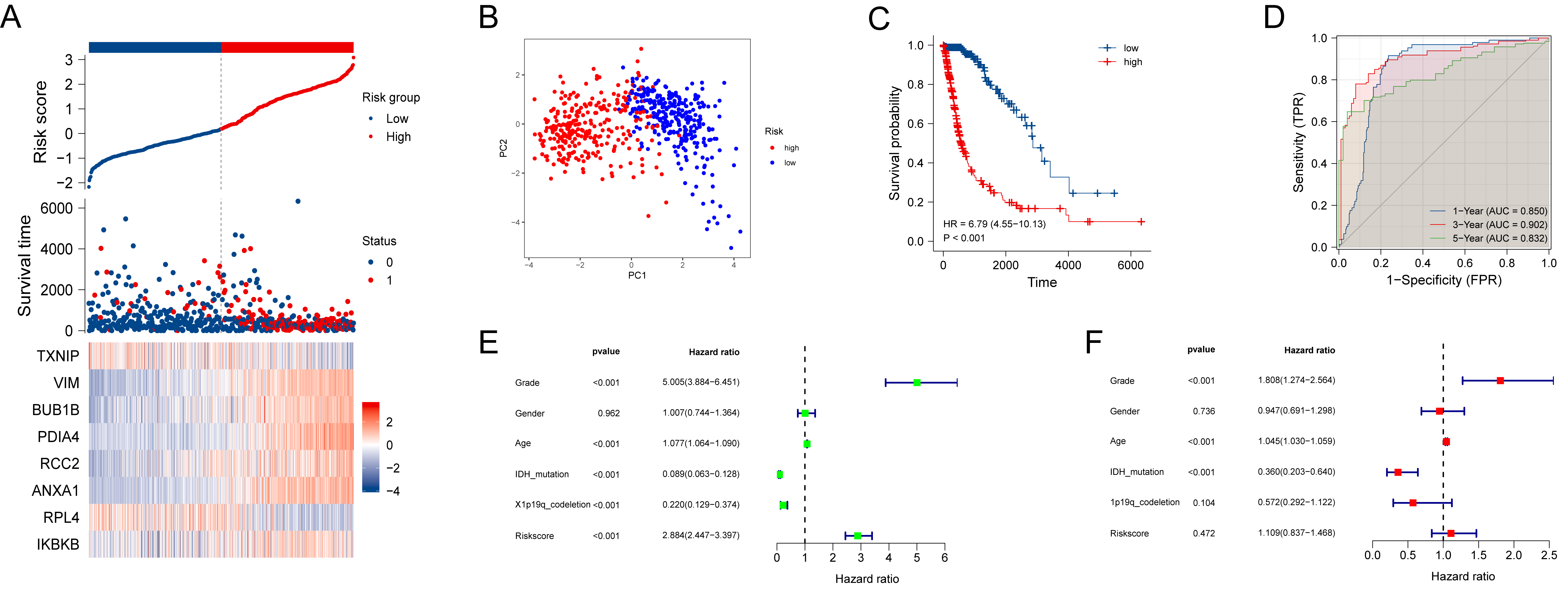

Supplement: Supplementary file 2 [file Image3.JPEG]

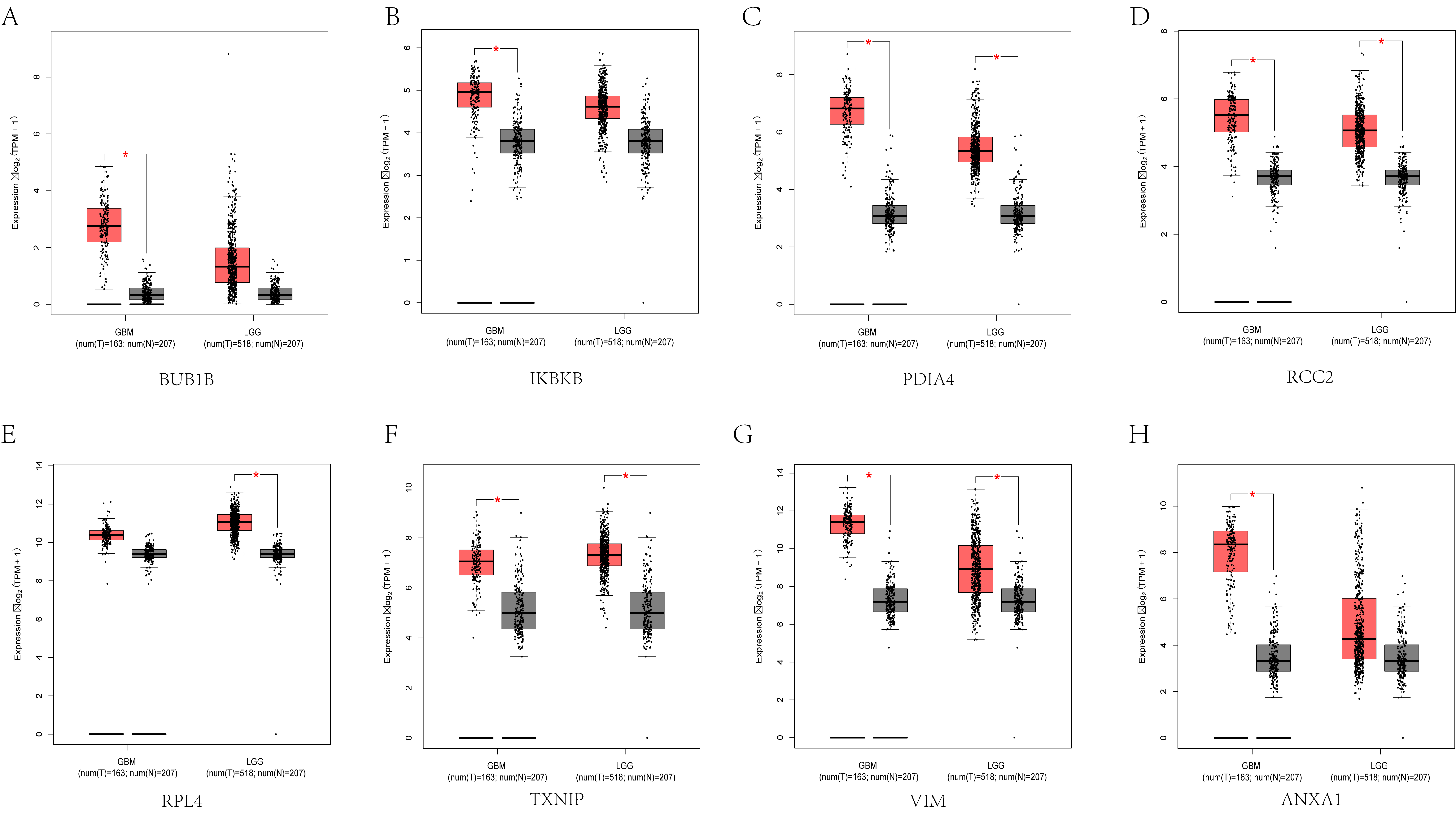

Supplement: Supplementary file 4 [file Image1.JPEG]

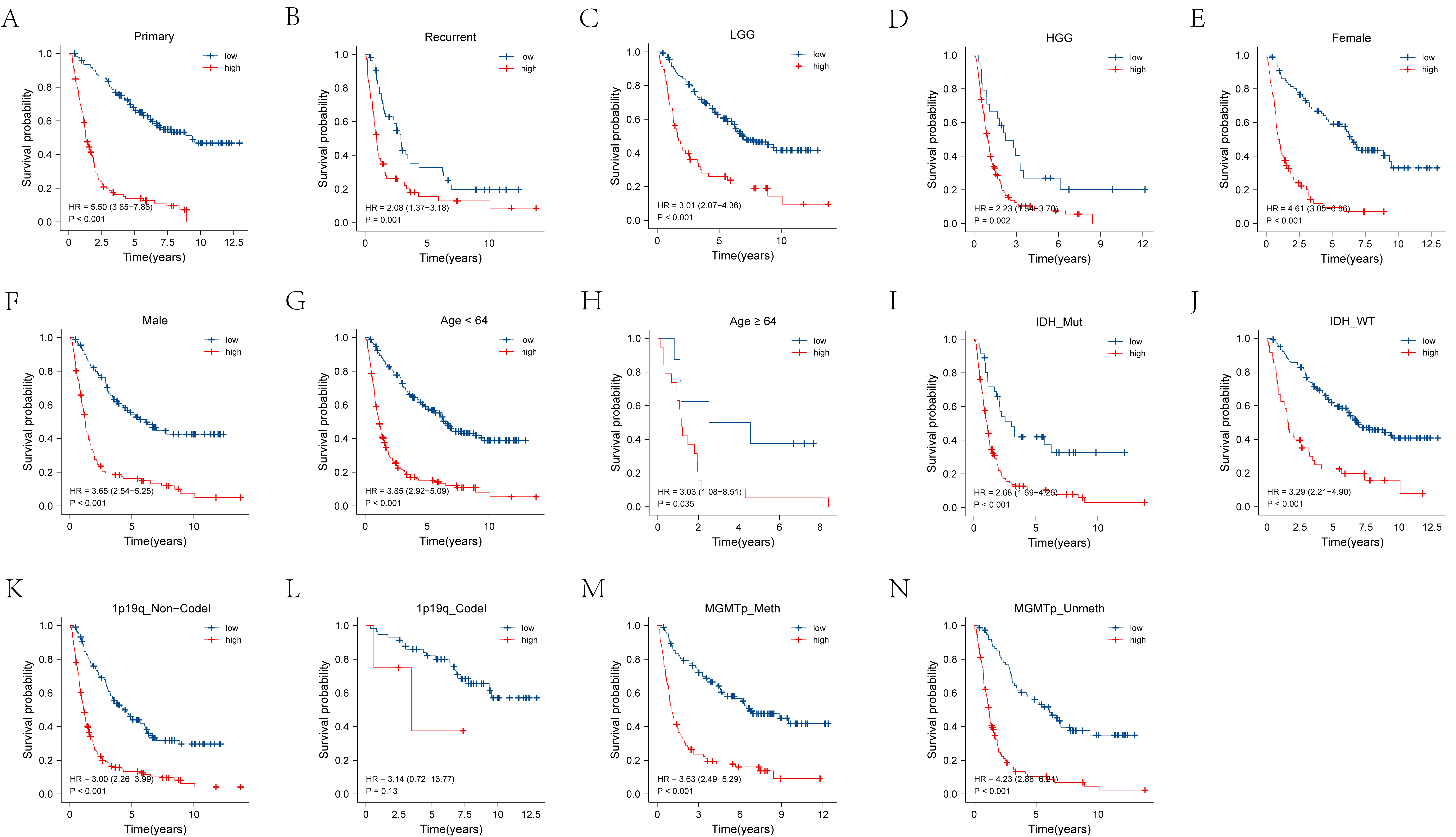

Supplement: Supplementary file 5 [file Image4.JPEG]

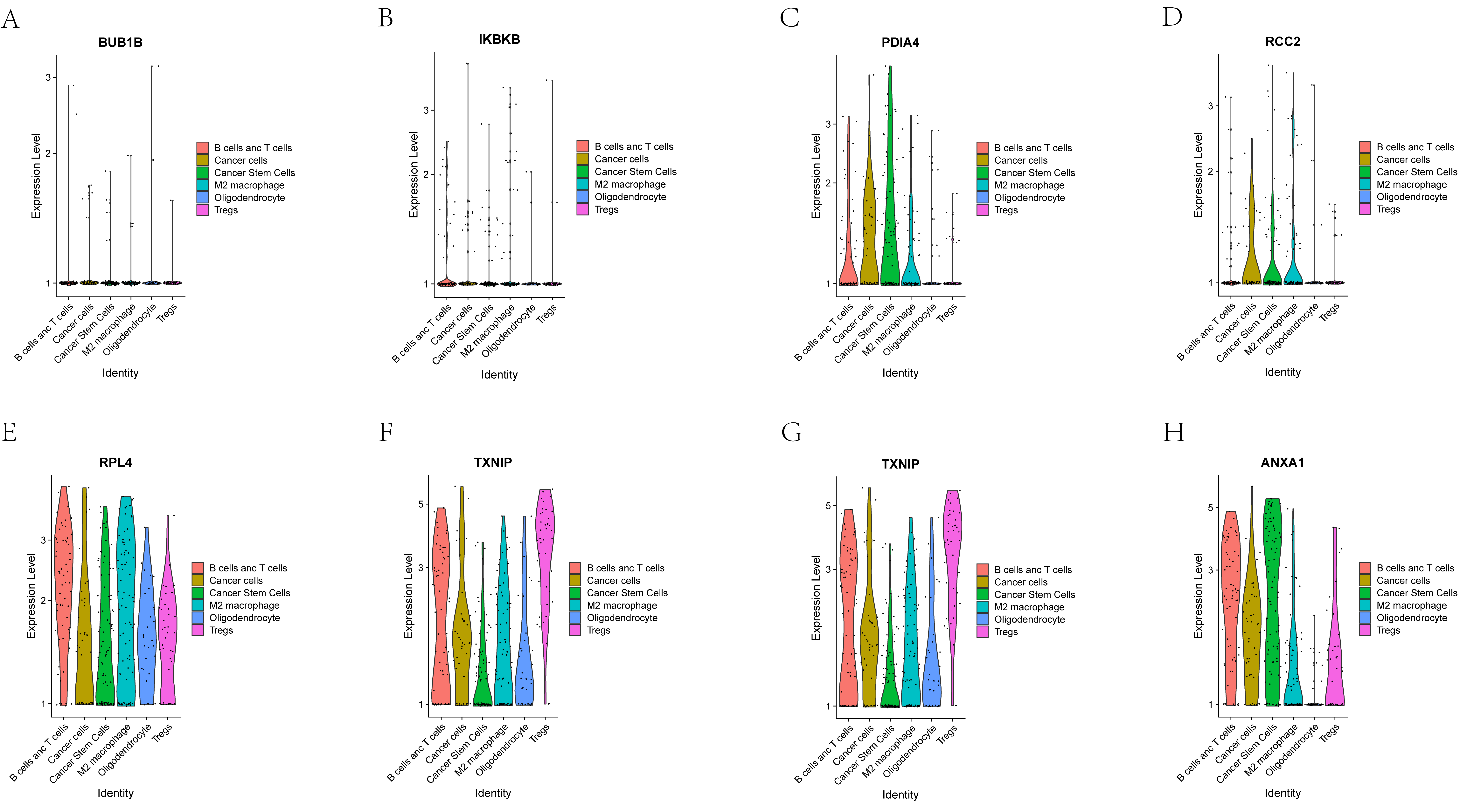

Supplement: Supplementary file 6 [file Image7.JPEG]

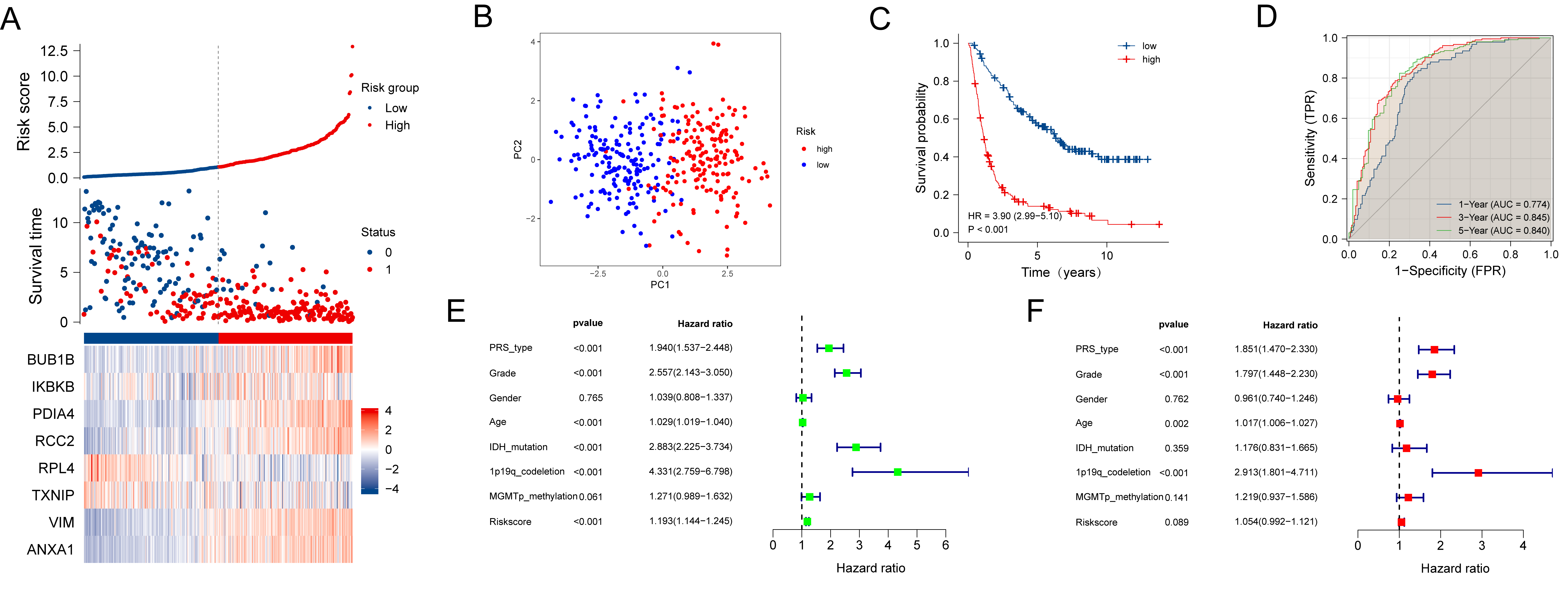

Supplement: Supplementary file 7 [file Image2.JPEG]

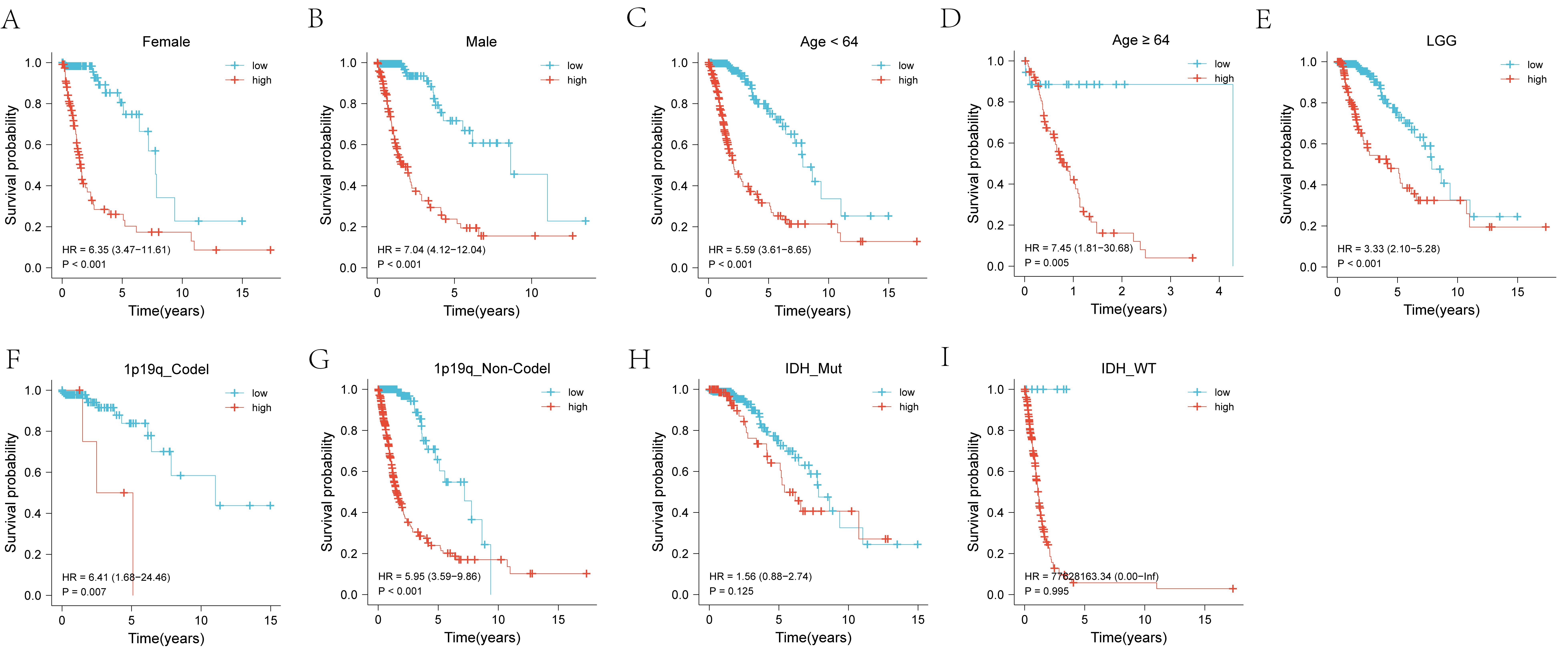

Supplement: Supplementary file 8 [file Image5.JPEG]

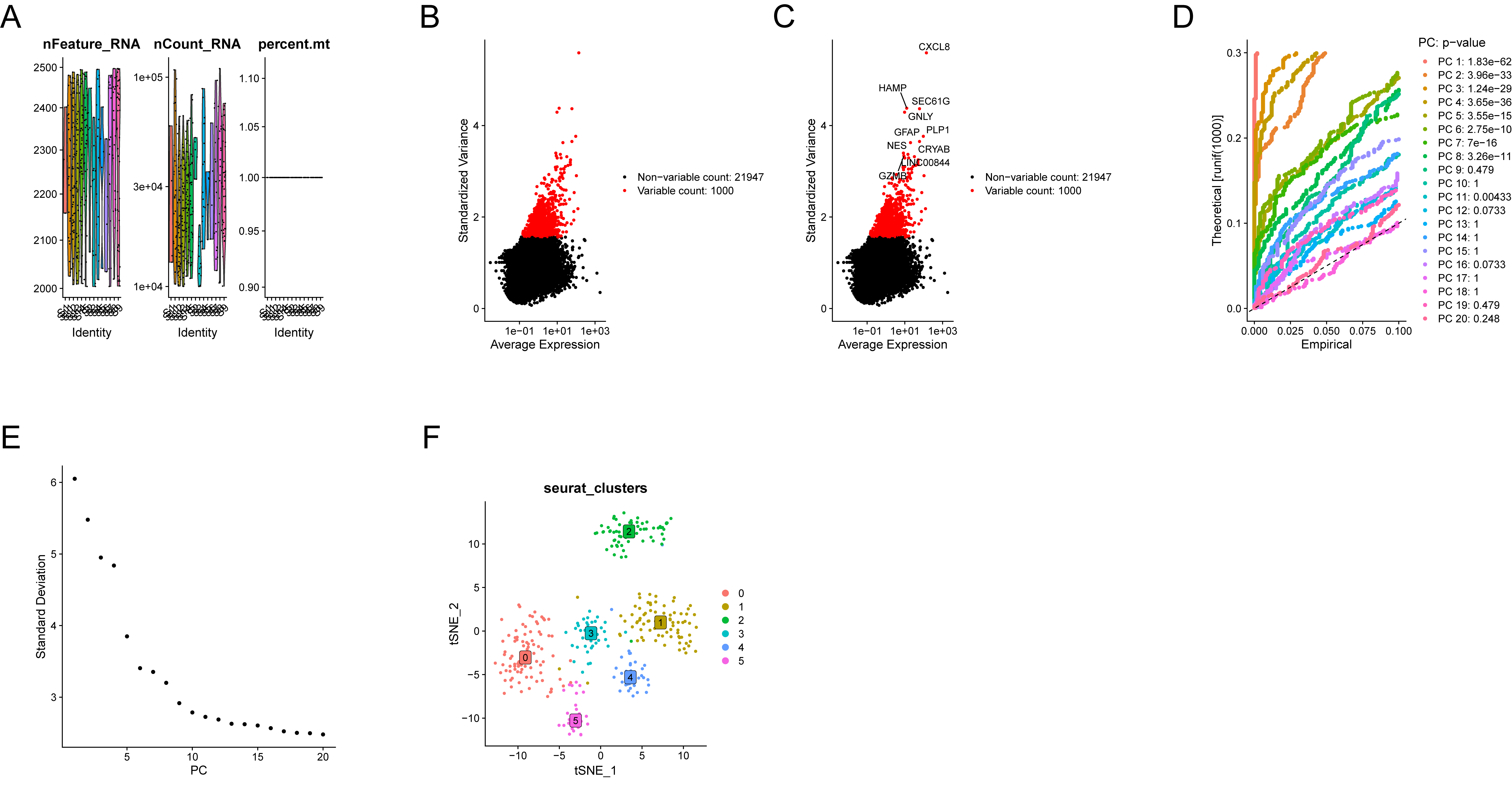

Supplement: Supplementary file 14 [file Image6.JPEG]
